# Combined Biopsy and Imaging-Guided Microwave Ablation by Using a Coaxial Guiding Needle

**DOI:** 10.5334/jbsr.2345

**Published:** 2021-05-07

**Authors:** Yi-Wei Wu, Gabriel Chan, Ivan Kuang Hsin Huang, Justin Kwan, Gavin Hock Tai Lim, Lawrence Han Hwee Quek, Uei Pua

**Affiliations:** 1 Tan Tock Seng Hospital, SG

**Keywords:** coaxial system, microwave ablation, percutaneous biopsy, thermal ablation, interventional oncology

## Abstract

This article demonstrates the technique of using a coaxial guiding needle to perform combined percutaneous biopsy and microwave ablation via a single tract. From May 2019 to July 2020, 14 patients underwent combined biopsy and microwave ablation by using a coaxial guiding cannula. Tumors were in the kidney of six patients (43%), the liver of six patients (43%), and the lung in two patients (14%). The diagnostic yield of biopsy was 86% (12/14). Ablation technical success rate was 100%. In conclusion, using a coaxial guiding needle in microwave ablation and biopsy is safe and effective.

## Introduction

In recent years, imaging-guided ablation has been used to treat tumors in different organs. Among several modalities of thermal ablation, microwave ablation (MWA) has been proven to be safe and effective in treating tumors in the liver, kidney, and lung [1,2,3].

Combined coaxial biopsy and ablation is such a common practice for radiofrequency ablation (RFA) [4] that an RFA device with an insulated guiding cannula is commercially available (LeVeen, Boston Scientific). At present however, there is no commercially available MWA system that provides a guiding cannula compatible with coaxial biopsy and MWA.

The aim of this article is to describe the technique of using a coaxial guiding needle to perform combined biopsy and MWA via a single path and to evaluate its feasibility.

## Technique

**Figure 1:**
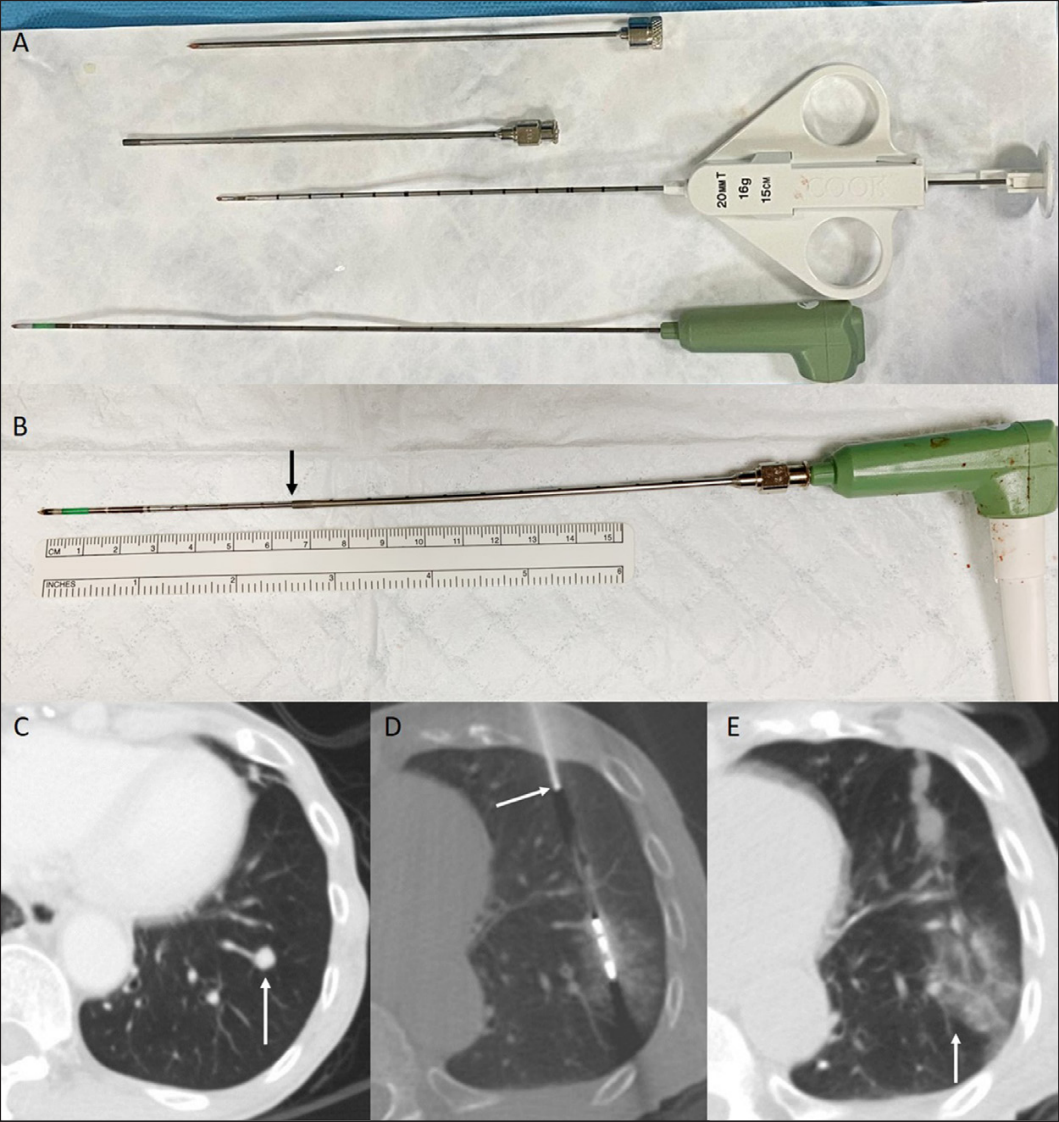
**(A)** Top to bottom: inner stylet of the guiding needle, 14G/11.6 cm guiding cannula, 16G biopsy needle and 17G/20 cm MWA antenna. **(B)** A MWA antenna inserted through guiding cannula. Tip of the guiding cannula is indicated by the arrow. **(C)** CT showed a 0.8 cm metastatic nodule in the left lower lobe. **(D)** Microwave ablation (MWA) was performed by MWA antenna inserted through the 14G guiding cannula (arrow). **(E)** Post procedure CT demonstrated satisfactory penumbra covering the nodule (arrow).

A 17G/20 cm MWA antenna (PR 20 probe, NeuWave Medical) can be inserted coaxially through the 14G/11.6 cm guiding cannula (***Figure 1A***).

During MWA procedure, a 14G/11.6 cm guiding needle was advanced into or near the tumor under ultrasound or computer tomography (CT) guidance. The inner stylet of the guiding needle was removed. Biopsy was performed with a 16G/15 cm needle coaxially through the guiding cannula. Subsequent MWA was performed with 17G/20 cm MWA antenna inserted coaxially through the guiding cannula (***Figure 1B***).

## Patients

A total 14 consecutive patients underwent combined percutaneous biopsy and imaging-guided MWA from May 2019 to July 2020.

Technical success is defined when tumor was treated according to MWA protocol of the chosen antenna and was covered completely by ablation zone immediately after the procedure. Complications were graded based on the Cardiovascular and Interventional Radiological Society of Europe (CIRSE) classification system [5].

Patients’ characteristics and procedure details are summarized in ***Table 1***. Overall diagnostic yield of percutaneous biopsy was 86% (12/14). Technical success was 100%. Of the 14 patients who underwent combined biopsy and MWA, two developed mild complications (14%).

**Table 1:** Patient characteristics, procedure details and outcome. F female, M male, US ultrasound, CT computer tomography, HCC hepatocellular carcinoma, RCC renal cell carcinoma.


NUMBER	AGE	SEX	ONCOLOGY HISTORY	LOCATION OF TUMOR	INDICATION OF BIOPSY	HISTOLOGY	MAXIMAL DIAMETER OF TUMOR (CM)	IMAGING GUIDANCE	NUMBERS OF ANTENNA	TECHNICAL SUCCESS	TRACT ABLATION OR EMBOLISATION	COMPLICATION, GRADE

1	90	F	Colonic adenocarcinoma	Liver, segment III	Confirm metastasis	Focal regeneration	0.8	US	1	Yes	No	No

2	64	M	No	Kidney, right upper pole	Confirm malignancy	Papillary RCC	1.5	CT	1	Yes	Tract ablation	No

3	53	F	Colonic adenocarcinoma	Liver, segment III	Confirm metastasis	Non-diagnostic	2.0	US	1	Yes	Tract ablation	No

4	78	M	No	Liver, segment VI	No risk factor for HCC	HCC	1.6	US	1	Yes	No	No

5	65	M	No	Kidney, left lower pole	Confirm malignancy	Non-diagnostic	1.8	US	1	Yes	Tract embolisation and ablation	No

6	68	M	No	Liver, segment V/VI	No risk factor for HCC	HCC	2.5	US	1	Yes	Tract ablation	No

7	59	F	No	Liver, segment VII	No risk factor for HCC	Focal lobular inflammation	1.0	CT	1	Yes	Tract ablation	No

8	76	M	No	Kidney, right upper pole	Confirm malignancy	Papillary RCC with sarcomatoid change	4.2	CT	2	Yes	Tract ablation	No

9	81	M	No	Kidney, left lower pole	Confirm malignancy	Clear cell RCC	3.5	US	2	Yes	No	No

10	72	M	No	Liver, segment III	No risk factor for HCC	HCC	1.6	US	1	Yes	No	No

11	79	M	No	Kidney, left upper pole	Confirm malignancy	Papillary RCC	3.0	CT	2	Yes	No	No

12	78	M	No	Kidney, right interlobar region	Confirm malignancy	Clear cell RCC with sarcomatoid change	3.4	CT	2	Yes	No	Ablation of psoas muscle with no clinical symptom, 1

13	86	M	Colonic adenocarcinoma	Lung, left lower lobe	Confirm metastasis	Metastatic adenocarcinoma	1.0	CT	1	Yes	Tract embolisation	No

14	79	M	Cholangiocarcinoma	Lung, left upper lobe	Confirm metastasis	Metastatic adenocarcinoma	0.6	CT	1	Yes	Tract embolisation	Small pneumothorax, resolved within 48 hours, 2


## Discussion

In our experience, combining biopsy and MWA through the same guiding cannula is relatively easy to perform. There are several advantages to using this technique. Firstly, biopsy is performed immediately prior to the placement of the MWA antenna. This allows the operator to better visualize and target the lesion, especially if the procedure is performed under ultrasound guidance. Using this technique, biopsy is performed by a needle with a larger diameter (16G), which may increase the diagnostic yield, as smaller needles (18G) are commonly used in tandem fashion. The biopsy diagnostic yield in our case series was 86%. Secondly, puncture-site complications (bleeding, pneumothorax, and tract seeding) are reduced, as the coaxial technique allows for performing two procedures via only one single path [4,6]. Lastly, the coaxial guiding cannula allows for injecting a hemostatic agent to embolize the tract once ablation is completed. This is particularly useful in lung ablation, as tract embolization may reduce pneumothorax rate after the procedure [7].

One of the limitations of using this technique is the risk of performing MWA on benign lesions if the histology result is not readily available. An additional limitation is that the biopsy of a small lung nodule may cause significant alveolar hemorrhage, which may hinder the subsequent placement of MWA antenna. To overcome this, the tip of the guiding cannula can be advanced over the biopsy needle to the distal margin of the nodule. Once the biopsy needle is removed, the tip of the MWA antenna is advanced to the tip of the cannula. Maintaining the position of the MWA antenna, the guiding cannula is slowly pulled back to expose the active tip of the MWA antenna. In that case, the tip of the MWA antenna is always at the distal margin of the nodule regardless of the presence of hemorrhage.

In conclusion, using a coaxial guiding needle in MWA was safe and effective in obtaining a biopsy prior to the MWA procedure via a single path.
